# Fatal Second Impact Syndrome in Rowan Stringer, A 17-Year-Old Rugby Player

**DOI:** 10.1017/cjn.2019.14

**Published:** 2019-05

**Authors:** Charles Tator, Jill Starkes, Gillian Dolansky, Julie Quet, Jean Michaud, Michael Vassilyadi

**Affiliations:** Canadian Concussion Centre, Toronto Western Hospital, Toronto, Ontario, Canada; Faculty of Medicine, University of Ottawa, Ottawa, Ontario, Canada; Faculty of Medicine, Memorial University of Newfoundland, St. John’s, Newfoundland, Canada; Faculty of Medicine, Dalhousie University, Halifax, Nova Scotia, Canada; Departments of Pediatrics, Emergency, Neuropathology, and Neurosurgery, Children’s Hospital of Eastern Ontario, Ottawa, Ontario, Canada

**Keywords:** Concussion, Adolescent athletes, Traumatic brain injury, Second impact syndrome

## Abstract

Second impact syndrome (SIS) is associated with malignant brain swelling and usually occurs in young athletes with one or more prior, recent concussions. SIS is rare and some dispute its existence. We report a case of SIS in Rowan Stringer, age 17, a rugby player who sustained a fatal brain injury despite prompt medical therapy including decompression surgery. The cause of the massive brain swelling was initially unknown. An inquest revealed Rowan’s text messages to friends describing symptoms from two prior, recent rugby brain injuries, likely concussions, within 5 days of the fatal blow and confirming the diagnosis of SIS.

## RÉSUMÉ

**Un cas de syndrome du second impact ayant entraîné le décès d’un joueur de rugby âgé de 17 ans.** Le syndrome du second impact (SIS) est associé à une forme maligne d’enflure du cerveau. Il se produit habituellement chez de jeunes athlètes victimes d’une ou de plusieurs commotions cérébrales récentes. On estime qu’un tel syndrome est rare ; certains en contestent même l’existence. Nous voulons rapporter ici un cas de SIS ayant affecté un joueur de rugby âgé de 17 ans : Rowan Stringer. Stringer est décédé à la suite de lésions cérébrales, et ce, en dépit de soins médicaux rapides lui ayant été prodigués, notamment une chirurgie de décompression. La cause de l’enflure massive de son cerveau est demeurée initialement inconnue. Toutefois, une enquête a révélé qu’il avait envoyé des messages texte à ses amis cinq jours avant le coup mortel. Dans ces messages, il décrivait les symptômes de deux lésions cérébrales survenues récemment, probablement des commotions cérébrales. Le contenu de ces messages texte vient ainsi confirmer le diagnostic de SIS.

The consequences of concussion can be severe, especially second impact syndrome (SIS), which causes severe neurological impairment or fatality.^1^ SIS is so rare that many clinicians never encounter a case, and there are uncertainties about its pathophysiology, with some experts doubting its existence.^2^ In SIS, there is at least one previous, recent brain injury such as a concussion and a second brain injury causing major neurological decline. The second injury is usually more severe and leads to malignant brain swelling which may be fatal, although may be successfully treated by early decompression therapy. SIS is preventable by preventing the second blow to the brain.

For unknown reasons, SIS occurs primarily in young athletes with sports being the most frequent venue for repetitive brain injury. Also unknown is why the brain swelling occurs so rapidly and profoundly. Possible mechanisms include loss of autoregulation and alteration of the blood–brain barrier.^1^^,^^3^

During an organized rugby game between two high schools in Ottawa, Ontario, Canada, on May 8, 2013, team captain Rowan Stringer, age 17, was high tackled, upended, and she landed on her head on the ground. She sat up momentarily then lost consciousness. The 911 call brought an Emergency Medical Services (EMS) team within minutes who found Glasgow Coma Score (GCS) was 3, fixed and dilated pupils (6 mm bilaterally), and spontaneous breathing. Intubation attempted at the field was unsuccessful due to trismus and the need for cervical spine precautions. Oxygen was administered (15 L/min) with bag and mask. Oxygen saturation was 100% during field assessment and resuscitation. Transport was on a backboard with cervical spine immobilization. During transport, there was another failed attempt at intubation, but throughout the initial assessment and transport, there was no hypoxia.

On arrival at the Children’s Hospital of Eastern Ontario, she had stable vital signs and was promptly intubated and ventilated. The right pupil became responsive following two doses of 3% hypertonic saline (4 mL/kg each). Throughout resuscitation, she remained hemodynamically stable: initial BP 120/70 with maximum of 131/93, no bradycardia, initial rate 85, and range 100–115.

**Figure 1:**
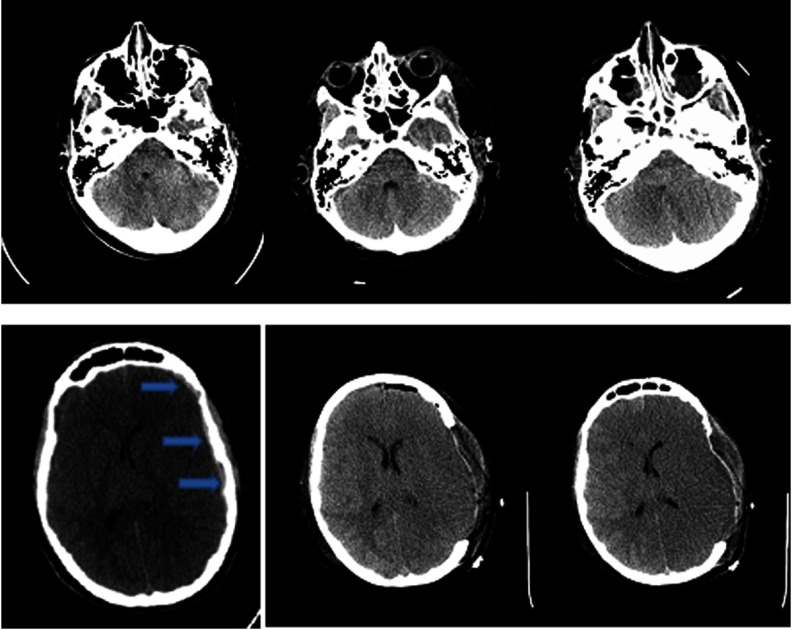
Non-contrast brain CT 2 hours after the patient’s injury (column A), 2 days post-injury (column B), and 3 days post-injury (column C). (A) There is generalized cerebral edema, moreso on the left side, with effacement of the sulci, uncal herniation, 3 mm midline shift to the right, and a small left-sided SDH approximately 2–3 mm thick (arrows). (B) Two days after left decompressive craniectomy with persistent generalized cerebral edema and interval resolution of the midline shift and signs of uncal herniation, and drainage of the SDH. (C) Progression of cerebral edema with complete absence of gray–white matter differentiation.

**Figure 2:**
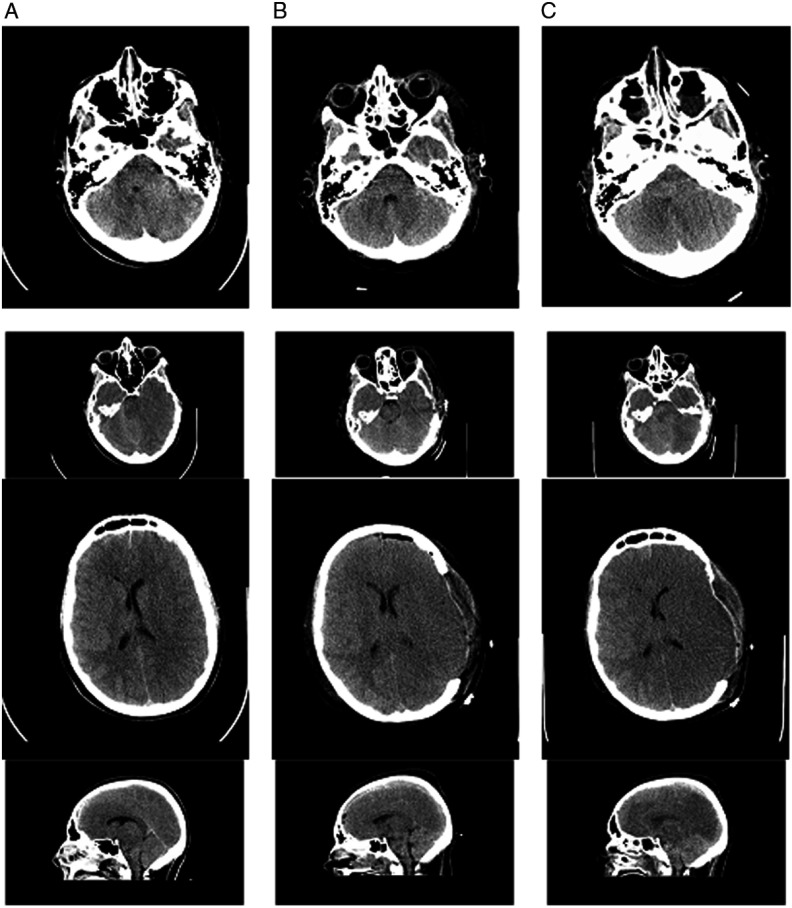
Additional Images of non-contrast CT head imaging 2 hours after the patient’s injury (column A), 2 days post-injury (column B), and 3 days post-injury (column C). There is initial generalized cerebral edema with early midline shift and a small left-sided SDH. The progression of cerebral edema and loss of gray–white matter differentiation, despite surgical and medical intervention including craniectomy, can be seen.

Brain and cervical spine CT scanning within 1 hour of presentation showed no fractures of the skull or cervical spine (Figure 1). There was significant, generalized cerebral edema, worse on the left with 3 mm midline shift to the right. There was a thin left subdural hematoma (SDH) about 2 mm in width over the convexity of the left cerebral hemisphere (Figure 1, column A). Mannitol was administered (∼1g/kg), and surgical decompression began 2.5 hours after presentation. A large, left decompressive craniectomy (9 × 14 cm) was performed, the SDH evacuated, and a parenchymal intracranial pressure (ICP) probe was placed. Mannitol was administered once intraoperatively, with no clear response. Initial post-op ICP was normal (5–8 mmHg), and the brain CT showed persistent edema with resolution of the midline shift and confirmed complete SDH removal (Figures 1 and 2, column B). Initial postoperative serum sodium was 149 mmol/L and peaked at 154 mmol/L 9 hours post-op. Urine output was normal, as per institutional Diabetes Insipidus (DI) protocol.

The first post-operative day, the patient remained unresponsive with ICP of 11–12 mmHg. The second day ICP was 12–14 mmHg, and DI developed (serum osmolality 320 mmol/kgH_2_0 and urine osmolality 141 mmol/kgH_2_0), and was stabilized by vasopressin infusion. The third day, ICP gradually rose to 30–35 mmHg and then spiked to 50 mmHg. The left pupil became fixed and dilated despite aggressive ICP management including four doses of hypertonic saline (3 mL/kg each), adequate pain control, muscle paralysis, normal BP and temperature maintenance, targeted low-normal pCO2 through careful ventilator management, and close monitoring of fluid balance. The brain CT showed worsening cerebral edema with compression of the ventricles, effacement of the cisterns, and suspected brainstem hemorrhage (Figures 1 and 2, column C). Three days post-op, the only brainstem reflexes were cough and spontaneous breathing, and the following day, gag and spontaneous respiration were absent.

After discussion with the family about the poor potential neurological outcome, the family decided to discontinue care. Neurological death was determined by clinical criteria 4 days after the final injury, and her parents agreed to perfused organ donation.

At autopsy 44 hours after death, the brain was harvested first, placed immediately in fixative, and showed severe cerebral edema. Brain weight was increased to 1449 g (normal: 1274±65 g). The left hemispheric convexity protruded through the craniectomy site, and there was herniation of both temporal unci at the tentorial hiatus and both cerebellar tonsils at the foramen magnum. There were no skull fractures. Serial sections showed no contusions or petechiae within the corpus callosum, deep white matter, or dorsal upper pons. Histologically, there was generalized hypoxic–ischemic encephalopathy (HIE) involving cerebral hemispheres, midbrain, pons, medulla, and cerebellum. There was generalized axonal damage indicative of diffuse axonal injury (DAI) of multifactorial etiology, including HIE, mass effect/herniations and acceleration/deceleration forces with scattered axonal swellings in the corpus callosum, dorsal brainstem, and cervical cord. Acceleration/deceleration injury was further supported by the acute SDH and a micro contusion of the left superior temporal cortex. Microscopy of the SDH removed at surgery confirmed fresh blood clots without evidence of subacute or chronic changes.

The neurosurgical team led by one of the authors (MV) was unable to explain the profound, immediate neurological decline present at the rugby field, followed by massive brain swelling causing further decline and death. Media reports of this case reached another author (CT) who had reviewed coroners’ reports of 631 deaths in sports and recreation,^4^ and agreed there was no apparent explanation for Rowan’s death. She died despite expeditious and expert care including the immediate 911 call from the rugby field, rapid EMS response, expert resuscitation, immediate transport to hospital, and a highly skilled hospital team providing rapid medical and surgical decompression of the brain. The small SDH could not have caused the massive brain swelling. The HIE was due to raised ICP with brain herniation at several locations. DAI can produce profound neurological damage but usually does not induce rapid, massive brain swelling.^5^^,^^6^ All authors agreed this rare and unusual case could be SIS, and that an inquest was needed to determine the diagnosis.

The inquest found the most exceptional and revealing evidence of SIS from Rowan’s own texted messages to and from her friends during the 5 days before her fatal injury. The detective assigned to the inquest retrieved Rowan’s cell phone and downloaded her texted messages. She texted about the blows to the head she sustained during a rugby game on May 3, 2013, and then again 3 days later on May 6, 2013, in a rugby game when she was “kicked in the head.” The texted symptoms including headache, fatigue, and tinnitus make it highly likely that she sustained concussions on May 3 and 6. The final fatal brain injury occurred on May 8, 2013, after which she never regained consciousness. The inquest found that between May 3 and 8, no adult diagnosed a concussion or was told about her May 3 and 6 concussions. As team captain, Rowan felt responsible, even obligated to keep playing while injured and still symptomatic from the previous injuries. She did not reveal her symptoms to any adult with whom she interacted in those 5 days of this tragedy. Tragically, she texted to friends that she had “googled concussion” and surmised that she “probably had a concussion.”

The cause of death determined at the inquest was SIS, and this conclusion was based on all the clinical, radiological, and neuropathological evidence.^7^ However, Rowan’s texted testimony of antecedent concussions was the most significant evidence. The imaging and pathological features of SIS are massive swelling of the brain causing brain herniation. These are nonspecific features that occur in many types of brain damage with accompanying raised ICP. There are only a small number of confirmed SIS cases, and all lacked the texted testimony of the deceased. It is noteworthy that almost all were young athletes, either teenagers or in their 20s, including the fatal, probable case in the 16-year-old Canadian hockey player reported as Case 1 in the report of 1968.^8^ Most cases die, but some survive because of emergency neurosurgical decompression providing extra space for the swollen brain. A minority had accurate descriptions of previous concussions that occurred days or weeks before the final catastrophic brain injury. Previous concussions may set the stage for the massive brain swelling that ensues.

**Figure 3:**
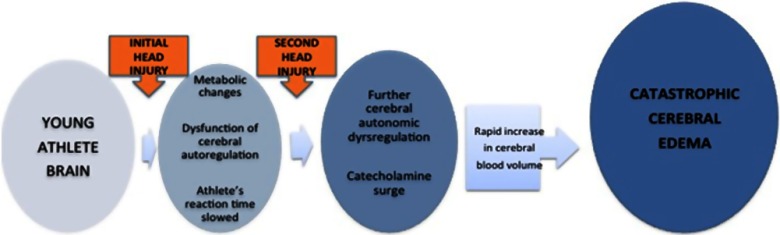
Proposed mechanisms of malignant brain edema in children and youth with concussion or mild traumatic brain injury. See text for explanation.^1^^,^^9^

The doubters of the existence of this syndrome label many with SIS as cases of first impact head injury.^3^ In both SIS and first impact head injury, damage to the brain can be caused by abnormalities of brain blood flow and brain blood vessel diameters, and by increased leakiness of blood vessels in the brain (Figure 3). In turn, these cause massive elevation of pressure in the brain and brain damage. Some term this malignant brain swelling.^3^ It is uncertain how the antecedent concussion makes the brain so vulnerable to another hit. Everyone in sports should know about SIS because it is preventable.
